# *Mycobacterium abscessus* urinary tract infection in an immunocompetent host: A case report and literature review

**DOI:** 10.1016/j.idcr.2022.e01538

**Published:** 2022-06-15

**Authors:** Abdulrahman F. Al-Mashdali, Gawahir A. Ali, Noheir M. Taha, Wael Goravey, Ali S. Omrani

**Affiliations:** a Department of Internal Medicine, Hamad Medical Corporation, Doha, Qatar; b Department of Infectious Diseases, Communicable Diseases Centre, Hamad Medical Corporation, Doha, Qatar; c Department of Laboratory Medicine & Pathology, Anatomic Pathology Section, Hamad Medical Corporation, Doha, Qatar

**Keywords:** *Mycobacterium abscessus*, Nontuberculous mycobacteria, Genitourinary infections, Chronic kidney disease, Clarithromycin, Amikacin

## Abstract

*Mycobacterium abscessus* is one of the nontuberculous mycobacteria (NTM), which can cause many clinical spectra, predominantly pulmonary infections followed by skin and soft tissue infections. The prevalence of *Mycobacterium abscessus* infections has been growing worldwide over the last two decades. Urinary tract infection (UTI) secondary to *M. abscessus* is a rare condition, and only five cases have been described in the literature so far. Therefore, managing such a condition is challenging and based on limited evidence. Here, we report a case of an adult male with a history of previous urological procedures who presented with lower urinary tract symptoms (LUTS) and was found to have a UTI secondary to *Mycobacterium abscessus*. In this case, we described our successful management approach of this rare entity of *Mycobacterium abscessus* infection, and we reviewed similar cases in the literature.

## Introduction

Nontuberculous mycobacteria (NTM) are a group of bacteria under the genus *Mycobacterium* including more than 172 species identified up to date. The prevalence of NTM infection is rising worldwide, especially in developed countries, which might reflect increasing awareness as a true pathogen with a significant advance in rapid and accurate identification of NTM from clinical specimens [1]. In addition, increasing the prevalence of immunocompromised hosts, including HIV, plays a role in NTM pathogenic potential [2]. The most common NTM species causing human diseases are the slow-growing *Mycobacterium avium* complex (MAC), which mainly affects the lung and associated with immunocompromised individuals [3].

*Mycobacterium abscessus* complex *(*MABC) infections are rapid grower, multi-drug resistant mycobacteria frequently associated with chronic pulmonary disease, traumatic skin wounds, surgical site infection, and healthcare-associated infections. Urinary tract infection (UTI) secondary to *M. abscessus* is an extremely rare condition [4]. To the best of our knowledge, only five *Mycobacterium abscessus* complex (MABC) UTI cases are reported in the literature [5], [6], [7], [8]. Herein, we report a complicated case of UTI secondary to *M. abscessus* in an immunocompetent adult, which was successfully treated with a long course of combined antimicrobial agents. Additionally, we reviewed the literature for similar cases.

## Case presentation

**Fig. 1:**
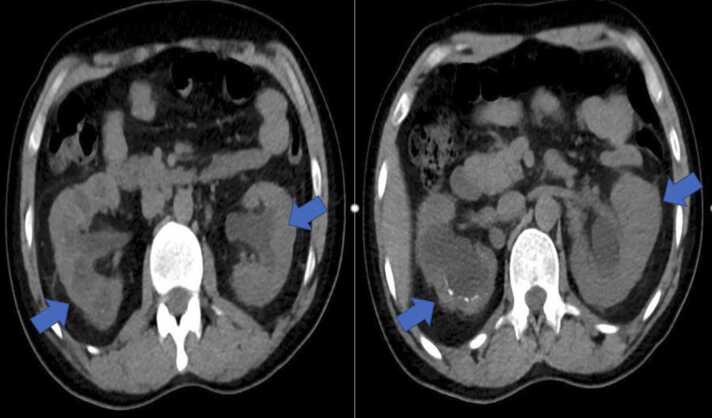
CT-KUB showing bilateral hydronephrosis with right kidney stones and perinephric fat stranding.

**Fig. 2:**
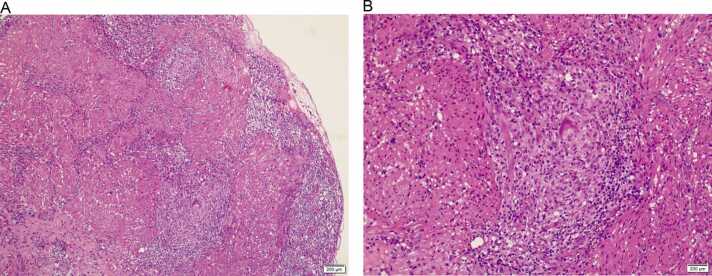
(A) Showing ulcerated urothelial mucosa with two non-necrotizing granulomas in the lamina propria consistent with granulomatous cystitis (Hx&E x 10); (B) showing non necrotizing granuloma with FB giant cell at the center surrounded by epithelioid cells and inflammatory cells (Hx&E x40).

A 37-year-old South Asian male was admitted to our hospital with dysuria and urine retention for one day. He has a history of stage 3 chronic kidney disease and bilateral obstructive uropathy due to kidney stones which required multiple invasive urological interventions three months ago prior to this presentation. Additionally, he was diagnosed with painful bladder syndrome requiring regular intravesical injections; the last one was four weeks before this presentation. On the following day of admission, he developed fever (temperature of 38.7 °C), and the laboratory investigations revealed white blood cells (WBC) of 7.1 × 10^3^/uL (normal range:4–10), C-reactive protein (CRP) of 93.7 mg/L(elevated), creatinine of 179 umol/L (within his baseline range), and urine WBC of 242/ hpf. Blood and urine cultures were negative. The urinary tract computed tomography revealed bilateral hydroureteronephrosis with no detectable ureteric or bladder stones (Fig. 1). Despite being on ertapenem (accordingly to his previous culture and sensitivity) for more than three days, he still had worsening urinary symptoms and spiking temperature. Repeated urine analysis revealed the presence of pus cells with negative cultures again. Accordingly, given the presence of recurrent sterile pyuria and his history of extensive urological interventions, a work-up for a mycobacterial UTI was performed. The Acid-Fast Bacilli (AFB) smears of the urine sample were negative. Cystoscopy with bladder biopsy was done, which revealed non-necrotizing granulomatous cystitis (Fig. 2). Two weeks later, urine culture(MTB cultures using both solid media, Middlebrook 7H11, and liquid media using BACTEC MGIT 960) grew *Mycobacterium abscessus* complex *(*MABC), which is later identified through real time PCR as *Mycobacterium abscessus* subspecies abscessus.

Subsequently, the patient was commenced empirically on a combination of meropenem (1 gm every 12 h), amikacin (433 mg once daily), ciprofloxacin (400 mg intravenous every 8 h), and clarithromycin (500 mg orally every 12 h). However, his creatinine worsened (increased from 170 to 249) due to amikacin nephrotoxicity; hence, amikacin was replaced by intravenous linezolid (600 mg every 12 h). However, two weeks after starting linezolid, the patient developed significant thrombocytopenia (platelet count dropped to 54), and subsequently, linezolid was substituted with doxycycline (100 mg oral every 12 h). The final urine culture and drugs susceptibility showed that the organism is resistant to doxycycline and ciprofloxacin and sensitive to tigecycline and clofazimine (Table 1). Therefore, he was kept on meropenem, tigecycline, clofazimine, and clarithromycin for two months, then meropenem and tigecycline stopped, and he was continued on clofazimine and clarithromycin. His monthly repeated urine mycobacterial cultures showed no growth, his HIV test was negative, and no abnormalities were detected in the chest- X-ray. At eight months follow-up from starting treatment, the patient was afebrile and asymptomatic, tolerating oral clofazimine and clarithromycin with persistent negative urine culture for mycobacterium abscessus. He was planned for 12 months of therapy from the first negative urine cultures.

**Table 1:** Antimicrobial susceptibility of the isolated MABC according to CLASI recommendations.

Antibiotics	MIC (mcg/ml)	Interpretation
Cefoxitin	32	I
Imipenem	4	S
Clofazimine	0.06	
Ciprofloxacin	> 4	R
Moxifloxacin	2	I
Clarithromycin	0.5	S
Amikacin	8	S
Tobramycin	8	R
Doxycycline	> 8	R
Tigecycline	0.12	
TMP/SMX	4/76	R
Linezolid	2	S

MIC = Minimum inhibitory concentration; S = Susceptible; I = Intermediate; R = Resistant

## Discussion

Mycobacterium abscessus complex *(*MABC) is classified as rapid-growing mycobacteria (RGM). The *M. abscessus group* is classified into three subspecies: *M. abscessus* subsp. *abscessus, M. abscessus* subsp. *bolletii,* and *M. abscessus* subsp. *massiliense*. These organisms are ubiquitous in the environment and can be found in soil, water sources, and decaying vegetation [4]. Historically, MABC was believed to be acquired exclusively from the environment. However, recent data from whole-genome sequencing (WGS) analysis of MABC *have* suggested a possibility of human-to-human transmission [9]. MABC is typically non-pathogenic but evolves as opportunistic infections that can lead to a wide range of infections due to the ability of MABC to produce multiple virulence factors (VFs), which facilitate survival within the host and immune masking to escape detection [10].

Pulmonary disease secondary to MABC is most prevalent in patients with cystic fibrosis (CF), whereas disseminated infection usually occurs in immunocompromised patients. Soft tissue infections due to MABC are usually healthcare-related and attributed to open wounds contaminated with non-sterile medical materials, but they also can occur due to wound contamination with soil [11]. Genitourinary infections caused by NTM are infrequent, and only a few cases have been reported [5]. Even rarer, genitourinary infections related to MABC have been identified [5], [6], [7], [8]. Our patient has a history of multiple admission and previous invasive urological instrumentation, which supports the hypothesis that invasive procedures can increase the risk of acquiring NTM related genitourinary infections [12]. Furthermore, outbreaks of M. abscessus complex infections in hospital settings have been reported worldwide, including contact transmission between patients [13].

Given its rarity, the data regarding UTI secondary to M. abscessus is limited [5], [6], [7], [8]. Based on a retrospective study of 15 patients with NTM genitourinary infections, chronic kidney disease (CKD) was the most common associated pre-existing condition. Also, characteristic urinary tract symptoms were documented in the majority of the patients(80%), including dysuria, urgency, flank pain, and hematuria. Interestingly, compared with urogenital mycobacterial tuberculosis, NTM related genitourinary infections were more likely to have constitutional symptoms, particularly fever. Notably, acid-fast smears of urine were negative in all patients [5].

On the other hand, the chest radiographs were abnormal in 27% of patients with genitourinary NTM infections, unlike 50–75% of those in urogenital mycobacterial tuberculosis [5]. Accordingly, the diagnosis of NTM genitourinary infections should be based on the culture of the drainage material or biopsy of the affected site. These observations were evident in our case, as he had CKD, constitutional symptoms, normal chest radiograph, and negative AFB smears in the urine. The persistent presence of sterile pyuria was the hint to investigate other potential possibilities and subsequently led to the diagnosis of MABC related genitourinary infection.

An accurate and reliable method is required for correctly identifying MABC to the subspecies level because significant interspecies differences exist in clinical relevance, pathogenicity, and antimicrobial susceptibility [14]. The Matrix-assisted laser desorption ionization (MALDI) TOF MS demonstrates the ability to distinguish M. chelonae and MABC but cannot differentiate between the three subspecies of MABC [15]. Therefore, multiple gene targets based sequencing, namely poB, gyrB, heat shock protein (hsp65), internal transcribed spacer (ITS), superoxide dismutase (sodA), and 16–23 S rRNA gene spacer amplification, are a reliable method for correctly identifying MABC to the subspecies level [15], [16].

*MABC is* associated with biofilm formation, resistance to disinfectants, high temperatures, and acidic environments [14]. Moreover, MABC is notoriously resistant to standard antituberculous agents and most antimicrobial agents. The natural resistance of MABC is related to its slow-growing nature, the permeability barrier of the complex multilayer cell envelope, drug export systems, enzymes that neutralize antibiotics, and genetic polymorphism of targeted genes. Furthermore, acquired resistance to aminoglycosides and macrolides is conferred by mutations affecting the genes encoding the antibiotic targets rrs and rrl, respectively [17]. Also, the erm(41) gene in MABC confers macrolide resistance through methylation of 23 S ribosomal RNA and can lead to inducible macrolide resistance and treatment failure if not routinely tested [14]. The Clinical and Laboratory Standards Institute (CLASI) recommends testing RGM for susceptibility to clarithromycin, amikacin, tobramycin, ciprofloxacin, moxifloxacin, imipenem, doxycycline, tigecycline, cefoxitin, cotrimoxazole, and linezolid [18]. The standard susceptibility testing method is broth microdilution, with the best in vitro antimycobacterial activity reported for clarithromycin, amikacin, and cefoxitin [19]. However, local susceptibility data are needed to guide treatment.

The optimal treatment for MABC- related genitourinary infections is not yet well defined, given its rarity. Thus, treatment recommendations rely on retrospective case series, and many areas need to be further studied and explored. In particular, optimal antimicrobial agents, optimal treatment duration, best combination therapy, the introduction of novel antimicrobial agents, and surgical intervention [17]. Treatment of MABC- related genitourinary infections requires 4–18 months of multi-drug therapy, which usually involves a macrolide-based regimen with two parenteral agents for the initial phase (Intravenous agents for two weeks to four months) followed by oral macrolide–based therapy [20]. Amikacin, cefoxitin imipenem, and tigecycline are the typical Intravenous treatment regimens. However, drug-related toxicity limits the use for intending period and jeopardizes the optimal management as described in our case. Follow-up MABC cultures conversion is crucial to ensure the response to the chosen regimen and guide the therapy duration [21].

We searched the PubMed databases in March 2022 for similar cases. The search was restricted to articles written in English and yielded 5 cases of MABC- related genitourinary infections (Table 2). Cases ranged from 22 to 74 years of age, and all were male. Only one patient had CKD as our patient [5], while almost two-thirds had lower urinary symptoms, although the data were not obtainable in two cases [5]. Only two patients reported positive urine AFB smears [7], [8]. Imipenem-cilastatin and amikacin were mainly used as injectable agents in 80% of the cases [5], [6], [8]. Of the cases identified, two were associated with prostatic abscess and drainage was required [5]. The duration of therapy ranged from 4 to 18 months, although data were not always available. All cases recovered completely from the infection.

**Table 2:** Summary of previously reported cases of *Mycobacterium abscessus* related Genitourinary infections.

Author/year of publication	Gender/Age	Comorbid condition	Initial presentation	Urine AFB	Antimicrobials	Duration of therapy	Surgical treatment	Outcome
Huang et al., 2010	63/M	CKD	N/A	-ve	Clarithromycin, imipenem-cilastatin, amikacin	N/A	Drainage of prostatic abscess	Recovered
Huang et al., 2010	74/M	None	N/A	-ve	Clarithromycin, imipenem-cilastatin, amikacin	N/A	Drainage of prostatic abscess	Recovered
Fongoro and Diallo, 2016	22/M	HTN	Uremic symptoms	+ve	Clarithromycin	N/A	None	Recovered
Laudelino et al., 2019	50/M	None	LUTS	N/A	Clarithromycin, amikacin	18 months	None	Recovered
Abolghasemi et al., 2021	62/M	None	LUTS	+ve	Clarithromycin, imipenem-cilastatin, amikacin	? 4–6 months	None	Recovered
Our case, 2022	37/M	CKD	LUTS	-ve	*meropenem, tigecycline, clofazimine, clarithromycin*	Still ongoing therapy (finished 8 months)	None	Recovered

## Conclusion

MABC- related Genitourinary infections in an immunocompetent host is a rare clinical entity with a paucity of clinical data regarding clinical management. Complex molecular techniques are needed to differentiate MABC to subspecies level, and well-designed studies comparing different regimens of antimicrobial agents are needed to determine the best treatment options. Meantime, careful clinical judgment is needed when encountering MABC- related Genitourinary infections to avoid organs destruction and detrimental consequences. The antimicrobial sensitivity pattern should guide treatment, and the optimal treatment duration remains unknown, but 4–18 months is suggested.

## Ethical approval

Ethical approval was obtained from Medical Research Centre (MRC) in Hamad Medical Corporation (HMC).

## Funding

This research did not receive any specific grant from funding agencies in the public, commercial, or not-for-profit sectors.

## CRediT authorship contribution statement

**Abdulrahman F. Al-Mashdali**: Acquisition of data, Drafting the manuscript, Approval of the version of the manuscript to be published. **Gawahir A. Ali**: Patient care, Drafting the manuscript, Approval of the version of the manuscript to be published. **Noheir M. Taha**: Provide the histopathology slides, Approval of the version of the manuscript to be published. **Wael Goravey**: Analysis and interpretation of data, Revising the manuscript critically for important intellectual content, Approval of the version of the manuscript to be published. **Ali S. Omrani**: Revising the manuscript critically for important intellectual content, Approval of the version of the manuscript to be published.

## Declaration of Competing Interest

The authors have no conflict of interest to declare.
